# Pneumomédiastin au cours d'une dermatomyosite, une entité rare: à propos d'un cas

**DOI:** 10.11604/pamj.2016.23.40.7986

**Published:** 2016-02-12

**Authors:** Senda Majdoub, Houcem Zemni, Houneida Zaghouani, Halima Houda Ben Salem, Habib Amara, Dajla Bakir, Chakib Kraeim

**Affiliations:** 1 Department of Radiology, University Hospital Farhat Hached, Sousse, Tunisia; 2 Department of pneumology, University Hospital Farhat Hached, Sousse, Tunisia

**Keywords:** dermatomyosite, complication, pneumomédiastin, dermatomyositis, connectivity, pulmonary complications, pneumomediastinum

## Abstract

la dermatomyosite est une connective caractérisée par une inflammation du muscle squelettique associée à des manifestations cutanées caractéristiques. Leurs étiologies, encore méconnues, associent facteurs environnementaux et génétiques. Parmi les complications pulmonaires décrites, les pneumopathies interstitielles qui sont des complications fréquentes. D'autres complications sont plus rarement rapportées comme le pneumomédiastin. Nous rapportons une observation de pneumomédiastin avec dissection aérique sous cutanée massive survenus chez une patiente atteinte de dermatomyosite. Nous discutons, à la vue des données de la littérature de la fréquence, des causes et des mécanismes physiopathologiques de cette pathologie.

## Abstract (en)

Dermatomyositis is a connective characterized by inflammation of skeletal muscle with cutaneous manifestations. Their etiologies, still unknown, associate environmental and genetic factors. Among lung complications described, interstitial pneumopathies are common complications. Other complications are rarely reported as pneumomediastinum. We report a case of pneumomediastinum with aeric in massive skin dissection occurred in a patient with dermatomyositis. We will discuss the frequency, causes and pathophysiology of the disease according to the literature.

## Introduction

La dermatomyosite est une myopathie inflammatoire idiopathique associée à des manifestations cutanées caractéristiques [1]. Parmi les complications pulmonaires décrites le pneumomédiastin spontané est une complication rare, quelques dizaines de cas seulement ont été publiés dans la littérature [2].

## Patient et observation

**Figure 1:**
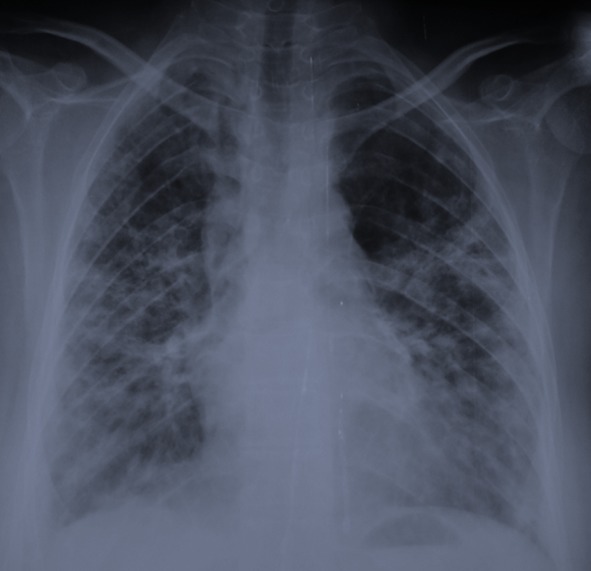
Radiographie thoracique de face: opacités réticulo nodulaires bilatérales en rapport avec un syndrome interstitial

**Figure 2:**
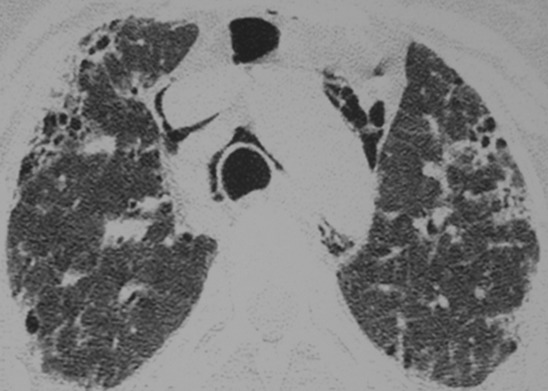
Scanner thoracique, coupe axiale passant pâr les apex pulmonaire: opacités réticulo no dulaires plutôt périphériques avec DDB par traction et de la fibrose sous pleurale

**Figure 3:**
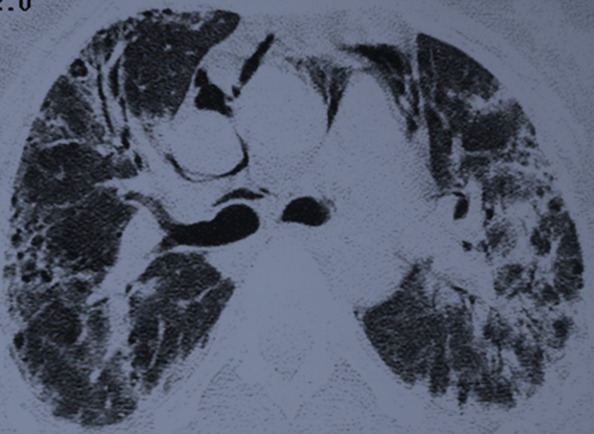
Scanner thoracique, coupe axiale passant par la carene: opacités réticulo nodulaires avec DDB par traction et de la fibrose sous pleurale. Il s’ y associe un pneumomédiastin de faible abondance plus marqué dans la région médiastinale antérieure et en péri tracheal

**Figure 4:**
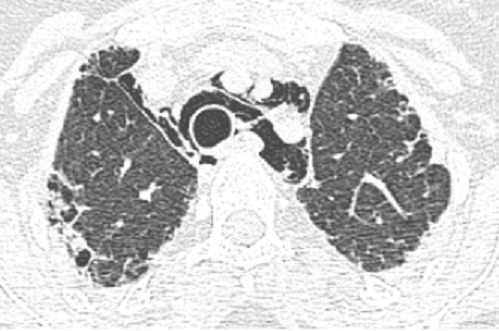
Scanner thoracique, coupe axiale passant au meme niveau au niveau des apex: aggravation du pneumomédiastin par rapport à l'examen precedent

**Figure 5:**
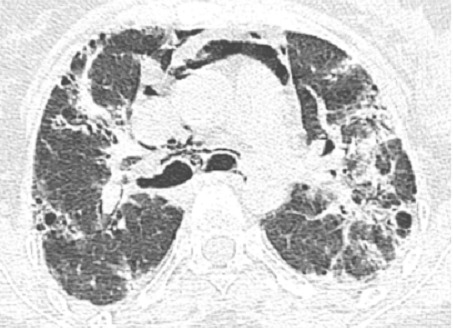
Scanner thoracique, coupe axiale passant par la carene: aggravation du pneumomédiastin et des des opacités réticulonodulaires

Madame H. 29 ans est hospitalisée dans le Service de Rhumatologie de notre centre hospitalier pour poussée d′arthralgies diffuses bilatérales et symétriques depuis 04 mois. Elles sont associées à des lésions érythémato papuleuses au niveau du dos des mains. Elle a reçu un traitement à base de corticothérapie à faible dose et de la nivaquine. L’évolution était marquée par l'apparition quelques mois après d'une asthénie progressive associée à une faiblesse musculaire et érythrœdème des paupières. Elle était adressée au Service de Dermatologie. L'examen a trouvé un œdème péri unguéal (signe de la manucure), des ulcérations en regard des articulations inter phalangiennes (papules de Gottron) un érythème héliotrope rose violacé des paupières et des plaques psoriasiformes au niveau des coudes. Ces différents éléments cliniques sont révélateurs d′une dermatomyosite débutante. Le bilan biologique retrouve un syndrome inflammatoire aspécifique, les enzymes musculaires ont des valeurs normales (Créatine Kinase = 33 U/l pour une norme < 170 U/l). L’électromyogramme trouve une atteinte myogène diffuse. Le bilan immunologique est normal. Les explorations fonctionnelles respiratoires retrouvent un syndrome restrictif modéré. Malgré l′absence de la totalité des éléments de la classification de Bohan et Peter en particulier la biopsie musculaire, le diagnostic de dermatomyosite est porté. L′échographie abdominale, la radiographie du thorax, la fibroscopie gastrique, la coloscopie, l'examen ORL et l′examen gynécologique ne retrouvent pas de néoplasie sous jacente. Au terme de ce bilan initial la patiente est mise sous corticothérapie et Imurel avec amélioration partielle de son asthénie et des lésions cutanés. Quelques mois après la mise sous traitement corticoïde d'entretien (faible dose), Madame H. présente de façon progressive une dyspnée d'effort stade II de MRC associée à une toux sèche et des douleurs basi thoraciques d'intensité modérée. L′auscultation pulmonaire trouve quelques crépitants au niveau des deux bases pulmonaires. Une radiographie de thorax de face a été réalisée montrant des opacités réticulo nodulaires bilatérales surtout basales et plus marquée du coté droit en rapport avec un syndrome interstitiel (Figure 1). La tomodensitométrie thoracique (coupes millimétriques en haute résolution sans injection de produit de contraste) réalisée pour une meilleure étude parenchymate use retrouvant les opacités réticulo nodulaires avec DDB par traction et de la fibrose sous pleurale. Il s’ y associe un pneumomédiastin de faible abondance plus marqué dans la région médiastinale antérieure et en péri tracheal ( Figure 2 et Figure 3). Un traitement symptomatique de la dyspnée est instauré puis la patiente est remise sous immunosuppresseur et corticothérapie à forte dose. L′évolution clinique est favorable avec une amélioration partielle des symptômes respiratoires mais le pneumomédiastin persiste de façon prolongée plusieurs mois après l′épisode initial. Lors d′un contrôle tomodensitométrique 02 mois après traitement, on objective la stabilité des anomalies parenchymateuses pulmonaires observées sur la première tomodensitométrie voir l'aggravation du pneumomédiastin qui s’étend vers les espaces péribronchovasculaires plus distales ( Figure 4 et Figure 5).

## Discussion

Les pneumomédiastins spontanés sont une entité rare associée souvent à un pneumothorax spontané. Ils peuvent être retrouvés au cours surtout de la dermatomyosite mais aussi dans un second plan des autres maladies systémiques notamment le lupus érythémateux disséminé, la sclérodermie et la polyarthrite rhumatoïde [2]. C'est Bradley en 1986 [3] qui décrit le premier cas de pneumomédiastin sur dermatomyosite et depuis, peu de cas ont été rapportés dans la littérature. Le point de départ d'un pneumomédiastin est la rupture d'une bronchiole terminale suite à un barotraumatisme. L'air libéré va donc suivre les structures péri broncho vasculaires vers les hiles pulmonaires, le médiastin puis les espaces cervicaux. L'entretien de cette issue d'air en dehors des voies aériennes peut être facilité par certains facteurs: -Les gluco corticoides sont considérés comme le principal facteur par leur effet nocif sur le tissu interstitiel pulmonaire, ce qui était le cas chez notre patiente. -Les micro organismes pathogènes (notamment les virus) dont la participation a été évoquée par Yamanishi [4]. La kystisation des lésions dues à la fibrose parenchymateuse pulmonaire avec commen conséquence des bulles d'air rappelant les blebs mais en situation endo parenchymateuse et non sous pleurale d'où l'absence de pneumothorax [5]. Les infarctus parenchymateux avec leur effet sur la paroi alvéolaire ont été évoqués par Cicuttini [6] étant donné la bonne évolution lors de la mise sous traitement immunosuppresseur. Lors d'une dermatomyosite, le diagnostic de pneumomédiastin spontané est évoqué devant une dyspnée avec une crépitation sous cutané notamment cervicale. La radiographie de face confirme généralement le diagnostic. Dans notre cas, vu que le pneumomédiastin n’était pas abondant, le recours à la tomodensitométrie thoracique en mode HR était nécessaire. En plus, cet examen permet de quantifier et localiser l'air extra pulmonaire. L’évolution est très variable. Dans notre cas il existe une stabilité radiologique de la lésion, associée à une amélioration clinique sous corticothérapie et immunosuppreseur. Parfois, l’évolution est rapidement fatale échappant à toute prise en charge thérapeutique [7].

## Conclusion

Nous avons rapporté un cas de pneumomédiastin spontané prolongé survenu dans le cadre d'une dermatomyosite. Son intérêt est de faire le point des mécanismes physiopathologiques expliquant la persistance, dans certains cas, du pneumomédiastin pendant plusieurs mois voir plusieurs années. L'apparition ou la modification des symptômes respiratoires doivent faire rechercher un pneumomédiastin et nécessiter une surveillance étroite à cause de la possibilité d'une évolution rapidement mortelle.
